# The breast cancer, early disease: toxicity from therapy with epirubicin regimens - cardiac assessment and risk evaluation (BETTER-CARE) study: CMR with early gadolinium relative enhancement, but not high-sensitivity troponin T, predicts the risk of chronic anthracycline cardiotoxicity

**DOI:** 10.1186/1532-429X-15-S1-O94

**Published:** 2013-01-30

**Authors:** Paul Kotwinski, Gillian Smith, Julie Sanders, Louise Ma, Jackie Cooper, Michael Thomas, Michael (Monty) G Mythen, Alison Jones, Hugh E Montgomery, Dudley J Pennell

**Affiliations:** 1 Institute of Human Health and Performance, UCL, London, UK; 2 CMR Unit, Royal Brompton Hospital, London, UK; 3 Oncology Department, UCL Partners, London, UK; 4 Department of Clinical Chemistry, The Royal Free Hospital, London, UK; 5 Biomedical Research Unit, UCLH/UCL, London, UK; 6 Centre for Cardiovascular Genetics, UCL, London, UK

## Background

A growing number of cancer patients are at risk from chronic anthracycline cardiotoxicity (cAC) as a result of improving cancer prognosis. Susceptibility is cumulative dose-related, but also idiosyncratic. At present there is no ideal test to identify those at risk: endomyocardial biopsy is inappropriate for routine monitoring; while serial measurement of LV ejection fraction (LVEF) only identifies cardiotoxicity after significant damage has been incurred. We hypothesised that risk of cAC could be determined from a combination of baseline factors and assessment of the response to the 1st anthracycline dose. Here we report the associations between cAC and myocardial insult after cycle 1: assessed by CMR and high-sensitivity troponin T (hsTnT).

## Methods

Women due to receive anthracycline-based chemotherapy for early breast cancer were recruited to the BETTER-CARE study. Those with known cardiovascular disease were excluded. CMR was performed before chemotherapy and at follow-up (>1 year after the final anthracycline cycle, and >3 months after Trastuzumab). A subgroup was studied at baseline and on day-3 after the 1st cycle of anthracycline with CMR (early gadolinium relative enhancement, EGRE) and measurement of hsTnT (Roche Diagnostics). A 2nd subgroup had hsTnT measured after the final anthracycline cycle (before radiotherapy or trastuzumab). LVEF was measured by a single operator (PK) and EGRE by a 2nd, independent operator (GS). Chronic AC (cAC) was defined as a fall in absolute LVEF≥5% at follow-up.

## Results

55/60 subjects assessed on day-3 completed follow-up (median 19 months); 51/55 had paired EGRE and 49/55 hsTnT data. 20% were in the cAC group (N=11). 58 subjects had hsTnT measurements after completing anthracycline treatment (median 21 days). No patients developed a rise in hsTnT after cycle 1. However, following the final cycle (median 4), 78% were hsTnT positive (≥0.004 μg/l). Post-treatment hsTnT was a poor discriminator of cAC (area under ROC curve of 0.51, p=0.95). The EGRE response was heterogeneous: median increase 11.6%, mean 23.8% (p<0.001). EGRE increased significantly more in those in the cAC group (p=0.02); area under ROC curve of 0.75 (p=0.01).

## Conclusions

This study shows that myocardial injury (elevated hsTnT) occurs in the majority of women treated with low-dose anthracyclines. However, hsTnT cannot discriminate between those who develop contractile dysfunction and those who do not. Myocardial inflammation can be detected after the 1st dose of anthracycline using CMR-EGRE, before hsTnT rises. Furthermore, the magnitude of EGRE response is greater in those who later develop contractile dysfunction due to cAC. Development of this, or related techniques, may lead to a superior means of monitoring the cardiotoxic effects of chemotherapy, both in clinical practice and interventional trials.

## Funding

This abstract presents independent research funded by the UK Department of Health under the Genetics Health Research Programme (PHGX23A). It was sponsored by University College London (UCL), and supported by the cardiovascular Biomedical Research Unit of Royal Brompton Hospital and Imperial College. All hsTnT assay materials were supplied by Roche Diagnostics (Mannheim, Germany). Patients were recruited through the auspices of the National Cancer Research Network (NCRN). The views expressed in this publication are those of the author(s) and not necessarily those of the NHS or the Department of Health.

